# Alpha-7 Nicotinic Receptor Dampens Murine Osteoblastic Response to Inflammation and Age-Related Osteoarthritis

**DOI:** 10.3389/fimmu.2022.842538

**Published:** 2022-04-08

**Authors:** Alice Courties, Juliette Petit, Ariane Do, Manon Legris, Inès Kouki, Audrey Pigenet, Pradeep K. Sacitharan, Francois-Paul Ehkirch, Francis Berenbaum, Jérémie Sellam

**Affiliations:** ^1^ Sorbonne Université, INSERM UMR 938, Centre de Recherche Saint-Antoine, Hôpital Saint-Antoine, Assistance Publique - Hôpitaux de Paris (AP-HP), Paris, France; ^2^ Department of Rheumatology, Assistance Publique - Hôpitaux de Paris (AP-HP), Saint-Antoine Hospital, Paris, France; ^3^ Institute of Ageing and Chronic Disease, University of Liverpool, Liverpool, United Kingdom; ^4^ Department of Biological Sciences, Xi’an Jiaotong-Liverpool University, Suzhou, China; ^5^ Groupe Maussins, Clinique des Maussins-Ramsay, Générale de Santé, Paris, France

**Keywords:** osteoblast, inflammation, osteoarthritis, nicotinic acetylcholine receptor (nAChR), aging

## Abstract

**Introduction:**

Osteoarthritis (OA) is a whole-joint disease characterized by a low-grade inflammation that is involved in both cartilage degradation and subchondral bone remodeling. Since subchondral bone has a cholinergic innervation and that acetylcholine (Ach) might have an anti-inflammatory effect through the α7 nicotinic Ach receptor (α7nAchR), we aimed (i) to determine the expression of non-neuronal cholinergic system and nicotinic receptor subunits by murine and human osteoblasts, (ii) to address the role of α7nAchR in osteoblastic response to inflammation, and (iii) to study the role of α7nAchR in a spontaneous aging OA model.

**Methods:**

Primary cultures of WT and α7nAchR knock-out mice (Chrna7^-/-^) murine osteoblasts and of subchondral bone human OA osteoblasts were performed. The expressions of the non-neuronal cholinergic system and of the nAchR subunits were assessed by PCR. *In vitro*, IL1β-stimulated WT, Chrna7^-/-^, and human osteoblasts were pretreated with nicotine. At 24 h, expressions of interleukin-6 (IL6) and metalloproteinase-3 and -13 (MMP), RANK-ligand (RANKL), and osteoprotegerin (OPG) were quantified by qPCR and ELISA. Spontaneous aging OA was evaluated and compared between male WT and Chrna7^-/-^ mice of 9 and 12 months.

**Results:**

Murine WT osteoblasts express the main components of the cholinergic system and α7 subunit composing α7nAchR. Nicotine partially prevented the IL1β-induced expression and production of IL6, MMP3, and RANKL in WT osteoblasts. The effect for IL6 and MMP was mediated by α7nAchR since nicotine had no effect on Chrna7^-/-^ osteoblasts while the RANKL decrease persisted. Chrna7^-/-^ mice displayed significantly higher cartilage lesions than their WT counterparts at 9 and 12 months, without difference in subchondral bone remodeling. Human OA osteoblasts also expressed the non-neuronal cholinergic system and α7 subunit as well as CHRFAM7A, the dominant negative duplicate of Chrna7. Nicotine pretreatment did not significantly reduce IL6 and MMP3 production in IL-1β-stimulated human osteoarthritic osteoblasts (*n* = 4), possibly due to CHRFAM7A.

**Conclusion:**

Cholinergic system counteracts murine osteoblastic response to IL-1β through α7nAchR. Since α7nAchR deletion may limit cartilage degradation during murine age-related OA, enhancing cholinergic system could be a new therapeutic target in OA but may depend on CHRFAM7A expression.

## Introduction

Osteoarthritis (OA) is the most prevalent joint disease characterized by joint pain and functional disability. Besides previous joint injury and obesity, a critical risk factor of OA is aging (1). Longer life expectancy may thus explain in part the current and future increasing incidence of the disease.

OA is mostly characterized by cartilage degradation but, as a whole-joint disease, subchondral bone remodeling and synovitis are also involved. The role of subchondral bone has been emphasized in many clinical as well as basic research studies demonstrating its involvement in established as well as in early OA (2). Subchondral bone undergoes several pathological changes including increased thickness of the cortical plate, osteophytes at the joint margin, remodeling of the trabeculae, and bone cysts. These changes involve both osteoclasts and osteoblasts. Osteoarthritic osteoblasts undergo excessive activation due to several types of stress such as excessive mechanical overload leading to the production of a range of pro-inflammatory mediators: cytokines such as interleukin-6 (IL-6), metalloproteinases such as MMP-3, lipid mediators, or alarmins (3–5). In addition, activated osteoblasts increase the release of receptor activator of NF-kappaB (RANK)-ligand (RANK-L) that eventually promotes bone resorption through the activation of osteoclasts (6). All these mediators participate to joint inflammation that further amplifies bone remodeling and subsequent cartilage degradation. Moreover, an increased number of vascular channels and microcracks in OA eases interactions at the bone–cartilage interface (7) explaining why osteoblast changes could also influence chondrocytes in the deep zone of articular cartilage (8).

The peripheral cholinergic system is defined by the production and metabolism of acetylcholine (Ach) and is mainly characterized by the vagus nerve. Beyond its parasympathetic role, vagus nerve stimulation has shown anti-inflammatory properties (9). Indeed, Ach exerts anti-inflammatory action mainly through its binding on the α7 nicotinic receptor (α7nAchR) expressed by a range of neuronal and non-neuronal cells (10). The α7nAchR is encoded by the Chrna7 gene. However, in human, Chrna7, native gene has been partially duplicated and rearranged on the same chromosome that is responsible for a dominant negative CHRFAM7A gene, which might decrease response to α7nAchR agonist because of the lack of agonist binding site (11).

Recently, using a 3-dimensional (3D) immunofluorescence analysis and tissue clearing protocol, we have shown that human OA subchondral bone receives a cholinergic innervation characterized by the expression of choline acetyltransferase (ChAT)-positive nerve fibers (12). Moreover, rat femoral metaphysis receives cholinergic (parasympathetic) innervation (13). Beyond the neuronal cholinergic systems, a range of non-neuronal cells are also able to produce and interact with Ach defining the non-neuronal cholinergic system (14). For example, chondrocytes belong to the non-neuronal cholinergic system since they express the molecular components to produce, transport, and degrade Ach as well as several nicotinic receptors including α7nAchR (15). α7nAchR activation can dampen inflammatory, degradative, and apoptotic responses of chondrocytes and could protect animals from OA (16–18). The α7nAchR involvement in bone mass has been evaluated in long cortical and trabecular bone while its involvement in the subchondral bone remodeling and on local bone inflammation has never been studied.

Considering the presence of cholinergic fibers and the possibility for a local non-neuronal cholinergic system in the subchondral bone, we aimed to determine whether human and murine osteoblasts are able to synthetize Ach and whether α7nAchR can dampen pro-inflammatory, pro-degradative, and pro-resorptive responses of stimulated osteoblasts as well as its involvement in Chrna7^-/-^ mice *in vivo*.

## Methods

### Animals

B6.129S7-Chrna7^tm1Bay/J^ knock-out (KO) Chrna7^-/-^ and their WT siblings mice were used for *in vitro* and *in vivo* experiments. All experiments were conducted in accordance with the Directive of the Council of the European Communities (2010/63/EU) on the Care and Use of Animals for Experimental Purposes and comply with the provisions of the French animal experimentation ethics committee “Charles Darwin” registered with the “Comité National de Réflexion Ethique sur l’Experimentation Animale”(Ile-de-France, Paris, No. 5). All procedures have been approved by the local ethics committee (No. 9173-20170330618083277). Mice were maintained in the animal facility under a standardized light–dark cycle and had free access to food and water.

### Knee Joint Assessment in Middle-Aged Mice

Male KO Chrna7^-/-^ and WT were sacrificed at 9 and 12 months for knee histological analysis. The right leg was dissected, fixed in 4% of paraformaldehyde (PFA), decalcified in ethylene-diamine-tetra-acetic acid (EDTA), and embedded in paraffin. Sagittal 5 μM sections of the knees were stained with Safranin-O/fast green. Cartilage degradation was scored according to the international OARSI score (sum of the tibia and femur score, each one scored on a 0–6 scale, so total score is 0–12, 12 being the worst) (19). For subchondral remodeling, knee sections pictured by Olympus DP73 camera were analyzed using Fiji. Subchondral bone was determined as the bone areas between articular and growth cartilages; we quantified the total bone area (T.Ar), the bone marrow area (Ma.Ar), and the trabecular bone area (B.Ar) (which is [T.Ar-Ma.Ar]). We reported the results as the ratio of Bone area/Total area (B.Ar/T.Ar) for tibia and femur separately.

### Murine Osteoblast Culture

Murine osteoblasts (OBs) were obtained from calvaria of 5-day-old WT and Chrna7^-/-^ litter as previously described (4, 20). Briefly, OBs were isolated by successive enzymatic digestions of Trypsin/EDTA (Sigma) for 20 min at 37°C and Liberase (Roche) at 0.8 U/ml at 37°C. After each digestion, cells were centrifugated (1,500 rpm, 5 min) and the pellet was re-suspended with DMEM (Dulbecco’s Modified Eagle’s Medium)-HAM F12 1 g/L glucose with 15 mM 4-(2-hydroxyethyl)-1-piperazine ethane sulfonic acid (HEPES) (Sigma) supplemented with penicillin 100 U/ml, streptomycin 100 μg/ml, L-Glutamine 2 mM (Ps-Glu), and 10% calf serum. After the last digestion phase, cells were seeded in a 12-well plate and cultured in DMEM-HAM F12, PS-Glu with CS 10% for 3 weeks. Successive addition of 50 μg/ml vitamin C and 5 mM β-glycerophosphate was performed during culture. The medium was changed every 48 h until the appearance of an extracellular matrix with calcifications, the main pattern of OB differentiation. The cells were then fasted for 24 h by replacing calf serum with 0.1% bovine serum albumin (BSA) before any stimulation.

### Primary Culture of Human Outgrowth OA Osteoblasts

Human OA subchondral bones were isolated from knee of patients undergoing joint total arthroplasty for OA at Saint-Antoine Hospital and Clinique Maussins Nollet - Ramsay Santé (Paris). Informed consent for the use of tissue was obtained from each patient before surgery. Experiments using human samples were approved by a French Institutional Review Board (Comité de Protection des Personnes, Paris Ile de France 5 and Commission Nationale de l’Informatique et des Libertés). According to a protocol developed by Hilal et al. and which we have adapted (21), human OA OBs are obtained from OA tibial plateau subchondral bone, and both sclerotic and non-sclerotic zones were used. Bones were first cut into 2-mm^2^ pieces before their successive enzymatic digestions by collagenase type I 1 mg/ml (Roche) at 37°C for 3 times (20 min, 20 min, and 4 h). After washing with phosphate buffered saline (PBS, Sigma), the digested bone fragments were cultured at 37°C in DMEM PS-Glu with 20% CS medium. The medium was changed every 48 h until adherent cells are observed. The bone pieces were then removed. At confluence, cells were seeded in 12-well plates (1 ml/well containing 30,000 to 50,000 cells) and the culture in DMEM PS-Glu and 10% of CS. At confluence, 2 mM proline, 10^-8^ M vitamin D3, and 50 μg/ml vitamin C were added to the medium for differentiation. The cells were maintained for about 15 days in this medium. They were then fasted for 24 h by replacing the SVF with 0.1% bovine serum albumin (BSA). To confirm osteoblastic differentiation, we did red alizarin staining. Briefly, cells were rinsed with PBS, fixed in 4% PFA, and incubated with 1% red alizarin solution (pH 4.2) for 20 min. Cells were then rinsed with water and pictures were taken. In addition, we evaluated the basal mRNA expression of 3 markers: collagen type I and osteocalcin (OB markers) and collagen type X marker (hypertrophic chondrocyte marker).

### Culture Treatment

Both murine and human OA OBs were stimulated after 24 h of fasting. Cells were first pretreated 15 min with nicotine (Sigma) to activate all nAchR at a dose of 1, 10, and 100 μM, and then stimulated with IL1β at 10 ng/ml (Peprotech) for 24 h. RNA and supernatants were recovered and frozen at −80°C.

### PCR

Human and murine OBs were lysed with BL-T buffer (BL cell lysis buffer + Thioglycerol). The mRNAs were recovered and extracted from the OB using Promega kit. Their concentration was determined using a spectrophotometer (NanoDrop). For each sample, 500 ng (murine) or 250 ng (human) of mRNA was reverse transcribed into complementary DNA [Omniscript RT kit (Qiagen)].

Cholinergic system components’ gene expressions (carnitine acetyltransferase [carAT], choline acetyltransferase [ChAT], vesicular Ach transferase [VAchT], acetylcholine esterase [AchE], butyrylcholinesterase [BchE], and choline transporter-1 [CHT1]) were determined by RT-PCR as previously published (16). RT-PCR products were subjected to 2% TRIS-acetate-EDTA agarose gel electrophoresis in TAE buffer using PCR products of cDNA coming from basal condition of murine and human OB. For each primer, water was used a negative control and spinal cord mRNA from donor bank was used as positive control.

Specific analysis of nAchR and of inflammatory, catabolic, and bone remodeling gene was performed by qPCR. We used the same primers as previously published for all cholinergic system components and tested the expression of cholinergic nicotinic receptors alpha (chrna1-7,9 and chrfam7a for human OB) and beta (chrnb1-4) (16). For qPCR, the cDNA is amplified using GoTaqPCR Master Mix Promega kit and measured in quantitative PCR (LightCycler Real Time PCR 480) using primers specific to the following genes: housekeeping gene 18S (human) or hypoxanthine-guanine phosphoribosyltransferase (HPRT) (murine), interleukin 6 (IL-6) gene, metalloproteinase (MMP) MMP-3 and MMP-13, nerve growth factor (NGF), osteoprotegerin (OPG), the receptor activator of nuclear factor kappa-B ligand (RANKL), and collagen type X, type I, and osteocalcine for human OB (Primers are listed in **Table 1**). Gene expressions were expressed as relative quantification of CT value on the housekeeping gene using the 2^−ΔCT^.

**Table 1:** List of human and mouse primers used for qPCR experiments.

	Human primers	
	Forward	Reverse
**18S**	5’-GCAATTATTCCCCATGAACG-3’	5’-GGGACTTAATCAACGCAAGC-3’
**IL6**	5’-CAATGAGGAGACTTGCCTGG-3’	5’GCACAGCTCTGGCTTGTTCC-3’
**MMP3**	5’-ATGAAAATGAAGGCTCTTCCG-3’	5’-GCAGAAGCTCCATACCAGCA-3’
**MMP13**	5’-GCCATTACCAGTCTCCGAGG-3’	5’-TACGGTTGGGAAGTTCTGGC-3’
**OPG**	5’-TGACTAATTTTGCCACAGGGTA-3’	5’-TGGATTAACCATTTGGGGTTT-3’
**RANKL**	5’-TGATTCATGTAGGAGAATTAAACAGG-3’	5’-GATGTGCTGTGATCCAACGA-3’
**Osteocalcin**	5’-ACC CAG GCG CTA CCT GTA TCA AT-3’	5’-AAA GCC GAT GTG GTC AGC CAA-3’
**Collagen type X**	5’-GCT TAC CCA GCA ATA GGA ACT CCC AT-3’	5’-CCA GTC CTT GGG TCA TAA TGC TGT-3’
**Collagen type I**	5’-GAC TGG CAA CCT CAA GAA GG-3’	5’-CAG GCT CCG GTG TGA CTC-3’
	**Mouse primers**	
	**Forward**	**Reverse**
**HPRT**	5’-AGGACCTCTCGAAGTGT-3’	5’-ATTCAAATCCCTGAAGTACTCAT-3’
**IL6**	5’-GTCACAGAAGGAGTGGCTA-3’	5-AGAGAACAACATAAGTCAGATACC-3’
**MMP3**	5’-TGAAAATGAAGGGTCTTCCGG-3’	5’-GCAGAAGCTCCATACCAGCA-3’
**MMP13**	5’-GATGGCACTGCTGACATCAT-3’	5’-TGTAGCCTTTGGAACTGCTT-3’
**OPG**	5’-ATCAGAGCCTCATCACCTT-3’	5’-CTTAGGTCCAACTACAGAGGAAC-3’
**RANKL**	5’-TGAAGACACACTACCTGACTCCTG-3’	5’-CCACAATGTGTTGCAGTTCC-3’
**NGF**	5’-CAC CCA CCC AGT CTT CC-3’	5’-CTC GGC ACT TGG TCT CAA A-3’

IL6, interleukin 6; MMP, metalloproteinase; HPRT, hypoxanthine-guanine phosphoribosyl transferase; OPG, osteoprotegerin; RANKL, receptor activator of nuclear factor kappa-B ligand; NGF, nerve growth factor.

### ELISA

After treatment of murine and human OB, the supernatants were recovered. IL-6, MMP-3, OPG, and RANKL were determined by ELISA using the Quantikine kit (R&D Systems) according to the manufacturer.

### Statistical Analysis

Unpaired non parametric *t*-test was used for OARSI scores and subchondral B.Ar/T.Ar ratio comparison between WT and Chrna7^-/-.^ For *in vitro* qPCR and ELISA, repeated measures (RM) one-way ANOVA was used for multiple groups comparison with Dunnett post-test to compare to IL1 beta alone condition. *p*-value <0.05 indicated statistical significance. Data are expressed as the mean ± standard error of the mean (SEM) or median [interquartile]. Statistical analysis was performed using GraphPad Prism 8 (GraphPad Software, San Diego, CA, USA).

## Results

### Non-Neuronal Cholinergic System Is Expressed by Murine Osteoblasts and Modulate Local Inflammatory, Catabolic, and Remodeling OB Activation to Inflammatory Stress

**Figure 1:**
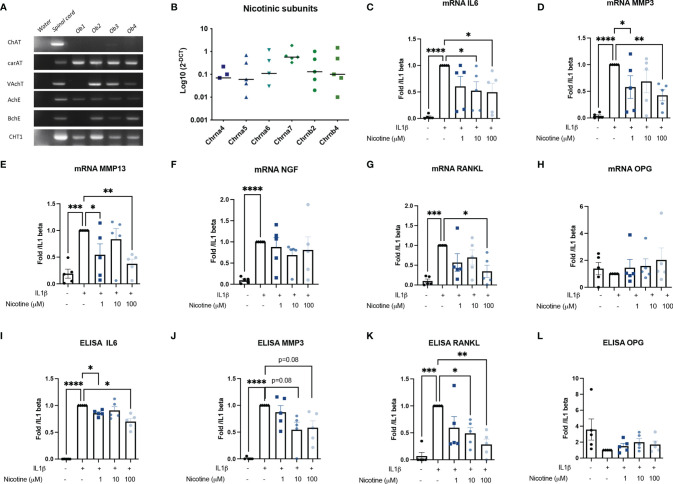
Murine OB expressed a cholinergic system and nAchR activation modulate response to inflammatory stress. **(A)** Representative RT-PCR analysis of the cholinergic system expressed by murine OB (*n* = 4 from Ob1 to Ob4). Water RNase-free condition is a negative control for each primer while mouse spinal cord mRNA is a positive control. **(B)** qPCR expression of nAchR subunits (*n* = 5). mRNA expression of **(C)** IL6, **(D)** MMP3, **(E)** MMP13, **(F)** NGF, **(G)** RANKL, and **(H)** OPG by WT OB pretreated with 1, 10, or 100 µM nicotine for 15 min and stimulated with 10 ng/ml IL1β for 24 h (*n* = 5 for WT, *p < 0.05 using RM one-way ANOVA with Dunnett post-test). The results are normalized to the IL1β condition. ELISA results of **(I)** IL6, **(J)** MMP3, **(K)** RANKL, and **(L)** OPG in WT OB pretreated with 1, 10, or 100 µM nicotine for 15 min and stimulated with 10 ng/ml IL1β for 24 h (*n* = 5 for WT, *p < 0.05 using RM one-way ANOVA with Dunnett post-test). The results are normalized to the IL1β condition. ChAT, choline acetyltransferase; carAT, carnityl acetyltransferase; VAChT, vesicular acetylcholine transporter; AChE, acetylcholine esterase; BChE, butyrylcholine esterase; CHT1, choline transporter-1; IL6, interleukin-6; MMP3, metalloprotease-3, NGF, nerve growth factor, OPG, osteoprotegerin; RANKL, the receptor activator of nuclear factor kappa-B ligand. *p<0.05, **p<0.01, ***p<0.001, ****p<0.0001.

Murine OBs belong to the non-neuronal cholinergic system since they expressed the main molecular members involved in the Ach synthesis (carAT) and transport (VAchT), Ach degradation (AchE and BchE), and choline precursor reuptake (CHT1) (**Figure 1A**). ChAT, the other enzyme involved in Ach production, was not expressed.

Moreover, OBs may be sensitive to cholinergic nicotinic agonists since they express many nicotinic receptor subunits: α4 (*n* = 3/5 experiments), α5, α6 (*n* = 4/5 experiments), and especially α7 as well as the β4 subunits. The expression of the α7 subunit predominates, although this was not significant (**Figure 1B**). Since all OBs express the α7 subunit, they are therefore potentially able to express the α7nAChR homopentameric receptor.

Stimulation of all nAchR by nicotine limited the osteoblastic response to IL1β. Pre-treatment of OB with nicotine reduced IL1β-induced IL6 mRNA expression by 47% for nicotine 10 μM and 51% at nicotine 100 μM (*p* < 0.05) and of MMP3 by 42% (not significant) for nicotine 10 μM and 58% at 100 μM (*p* < 0.05) (**Figures 1C–E**). We did not find an effect on mRNA NGF expression level (**Figure 1F**). Moreover, nicotine 100 μM decreased RANKL expression, while it had no effect on OPG mRNA (**Figures 1G, H, L**). This was confirmed at the protein level. Indeed, mean IL1-induced IL6 was 87,133 pg/ml with a maximum decrease to 47,495 pg/ml at 100 μM nicotine (mean decrease of 31%, *p* < 0.05 **Figure 1I**); mean IL1β-induced MMP3 was 2,558 ng/ml and decreased to a mean level of 1,352 ng/ml (mean decrease of 42%, *p* = 0.08, **Figure 1J**) at 100 μM nicotine, and IL1-induced RANKL was 400 decreasing to 161 pg/ml at 100 μM nicotine (mean decrease of 72%, *p* < 0.05) (**Figure 1K**).

### Alpha 7 Nicotinic Receptor Is Involved in Anti-Inflammatory and Anti-Catabolic Responses but Did Not Influence Bone Remodeling Markers *In Vitro*

**Figure 2:**
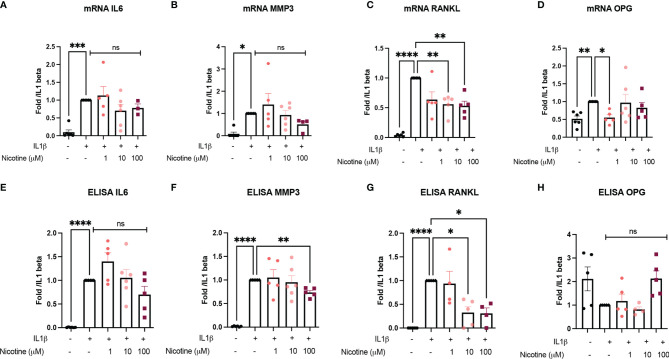
a7nAchR is involved in anti-inflammatory and anti-catabolic response *in vitro* but not bone remodeling marker. **(A)** IL6, **(B)** MMP3, **(C)** RANKL, and **(D)** OPG mRNA expression by Chrna7^-/-^ OB pretreated with 1, 10, or 100 µM nicotine for 15 min and stimulated with 10 ng/ml IL1β for 24 h (*n* = 5 for Chrna7^-/-^, *p < 0.05 using one-way ANOVA with Dunnett post-test). The results are normalized to the IL1β condition. ELISA results of **(E)** IL6, **(F)** MMP3, **(G)** RANKL, and **(H)** OPG in Chrna7^-/-^ OB pretreated with 1, 10, or 100 µM nicotine for 15 min and stimulated with 10 ng/ml IL1β for 24 h (*n* = 5 for WT, *p < 0.05 using RM one-way ANOVA with Dunnett post-test). The results are normalized to the IL1β condition. *p<0.05, **p<0.01, ***p<0.001, ****p<0.0001.

Because α7nAchR is known to be involved in inflammatory response, we investigated whether the anti-inflammatory and anti-catabolic effects were due to α7nAChR using OB from Chrna7^-/-^ mice performing similar experiments (nicotine pre-treatment and IL1β stimulation). We found that the nicotine effect on IL1β-induced IL6 and MMP3 mRNA and protein was clearly attenuated in Chrna7^-/-^ OB compared to WT (**Figures 2A–F**). In contrast, the effect on RANKL persisted, meaning it was independent of α7nAchR and might depend on other nAchR (**Figures 2C, D, G, H**).

### Alpha 7 Deletion Is Associated With More Cartilage Degradation Associated With Aging but Did Not Influence Subchondral Bone Remodeling *In Vivo*

**Figure 3:**
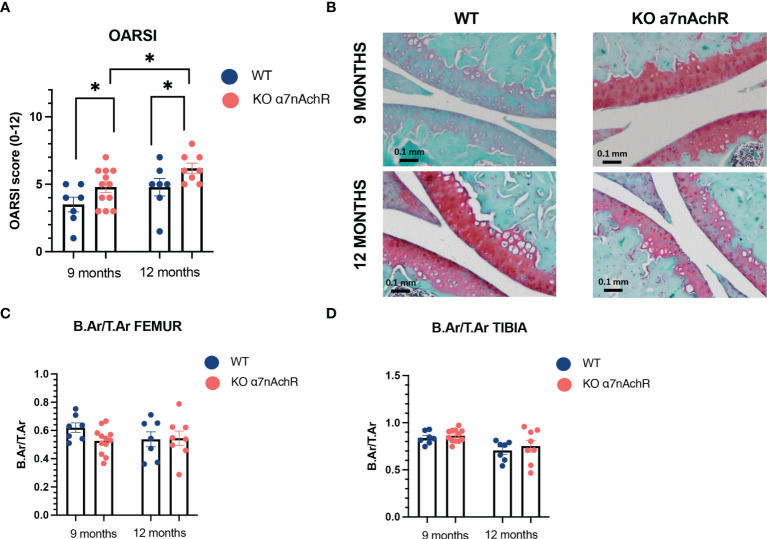
Cartilage and subchondral bone histological analysis in 9- and 12-month WT and Chrna7^-/-^ male mice. **(A)** OARSI score of the sum of femoral condyle and tibial plateau (0–12, 12 being the worst) between 9-month WT (*n* = 7) and Chrna7^-/-^ (*n* = 12) and 12-month WT (*n* = 7) and Chrna7^-/-^ (*n* = 8) male mice. **(B)** Representative Safranin O/fast green-stained sections of the right knee of 9- and 12-month WT and Chrna7^-/-^ mice. **(C)** B.Ar/T.Ar femur and **(D)** tibia subchondral bone of the 9-month WT (*n* = 7) and Chrna7^-/-^ (*n* = 12) and 12-month WT (*n* = 7) and Chrna7^-/-^ (*n* = 8) male mice, *p < 0.05 using non parametric *t*-test.

Considering the bone–cartilage unit in OA and communication between both tissues, we next wanted to evaluate both cartilage and bone modifications during mice aging in male WT and Chrna7^-/-^. *In vivo*, we observed slightly more cartilage lesions in Chrna7^-/-^ mice at 9 and 12 months compared to WT with a respective median [interquartile] OARSI score of 5 [3.25–6] in Chrna7^-/-^ and 4 [2.5–5] in WT at 9 months (*p* < 0.05) and of 6 [5.12–7] in Chrna7^-/-^ and 5 [4–6] in WT at 12 months (*p* < 0.05) (**Figures 3A, B**). However, we did not find substantial modification of subchondral bone remodeling between Chrna7^-/-^ and WT mice based on a similar Bone area/Total area (B.Ar/T.Ar) between both strain mice (**Figures 3C, D**).

### Cholinergic Anti-Inflammatory and Anti-Catabolic Response Is Decreased in Human Potentially Due to Dominant Negative Duplicate Expression

**Figure 4:**
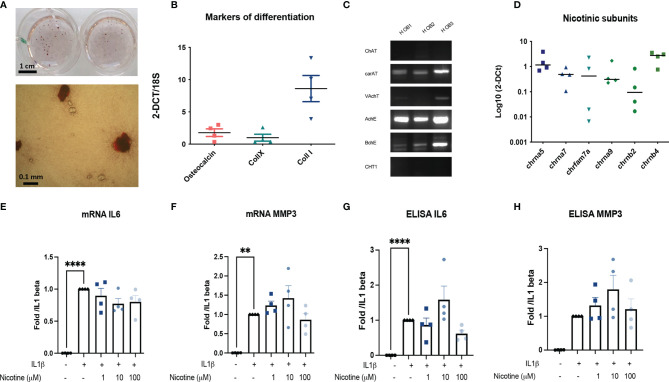
Primary human outgrowth OA OB belongs to the non-neuronal cholinergic system. **(A)** Representative calcification of extracellular matrix of Human OB using red alizarin staining. **(B)** Relative mRNA expression of osteocalcin, collagen type X (collX), and collagen type I (Coll I) of Human OB. **(C)** Representative RT-PCR analysis of the cholinergic system expressed by Human OB (*n* = 3). **(D)** qPCR expression of nAchR subunits (*n* = 4). **(E)** mRNA IL6, **(F)** MMP3 and ELISA, **(G)** IL6, and **(H)** MMP3 expression by Human OB pretreated with 1, 10, or 100 µM nicotine for 15 min and stimulated with 10 ng/ml IL1β for 24 h (*n* = 4, **p < 0.01, ****p < 0.001 using RM one-way ANOVA with Dunnett post-test). The results are normalized to the IL1β condition. ChAT, choline acetyltransferase; carAT, carnityl acetyltransferase; VAChT, vesicular acetylcholine transporter; AChE, acetylcholine esterase; BChE, butyrylcholine esterase; CHT1, choline transporter-1; IL6, interleukin-6; MMP3, metalloprotease-3; NGF, Nerve Growth Factor; OPG, osteoprotegerin; RANKL, the receptor activator of nuclear factor kappa-B ligand.

Then, we determined whether human OA OBs may also participate in local response to inflammatory stress through nAchR. Human OA OBs were characterized by the expression of collagen type I and calcification production (**Figures 4A, B**). They also belong to the non-neuronal cholinergic system since they expressed CarAT, AchE, and BcHE (**Figure 4C**) and nicotinic subunits with both native subunit Chrna7 and its human specific duplicate called CHRFAM7A (**Figure 4D**). However, *in vitro*, we did not observe any significant effect of nicotinic receptors activation on IL1β-induced IL6 and MMP3 in both mRNA and ELISA (**Figures 4E, F**), or on RANKL and OPG (not shown). Mean baseline IL1β-induced IL6 protein was 49,7334 pg/mL with no significant decrease with nicotine at any dose (**Figure 4G**) and mean baseline IL1β-induced MMP3 was 510 pg/ml with no decrease with nicotine (**Figure 4H**) (mean level of MMP3 production 517.5 pg/ml for IL1β with 100 μM nicotine for example).

## Discussion

This study suggests that murine OB belongs to the non-neuronal cholinergic system and that the nAchR activation of OB could partially counteract response to an inflammatory stress. The catabolic and inflammatory responses might be mediated by α7nAchR in murine OB while the effect on bone remodeling might be dependent on other nAchR. This was confirmed *in vivo* since middle-aged Chrna7^-/-^ mice show slightly more cartilage defects compared to WT with no modification in subchondral bone. Whereas human OA OBs also belong to the non-neuronal cholinergic system, the activation of nAchR did not influence their response to inflammatory stress. This discrepancy might be due to the presence of a specific dominant duplicate CHRFAM7A decreasing Ach response or to the expression of other nicotinic receptors containing Chrna5, Chrnb2, or b4 (11).

Non-neuronal cholinergic system is characterized by the presence of all the molecular components involved in Ach metabolism and response. We focused here on the main one and on nicotinic receptors. Previous studies have shown that the murine OB-like cell line and murine OB from calvaria expressed most of the components of the cholinergic system (22, 23) and that their expression might depend on the differentiation state. OB did not express ChAT, suggesting that the local OB non-neuronal production of Ach could be controversial. However, they expressed carnityl acetyltransferase (carAt), another enzyme, that has been shown to produce Ach in other non-neuronal tissues (denervated muscle and rat sperm) (24, 25). Additionally, since cholinergic fibers are present in human and rodent subchondral bone, the presence of nAchR might also be activated by the neuronal Ach locally produced within the joint (12, 13). Alpha-7nAchR has been involved in a large range of physiological processes in central nervous system as well as in inflammatory peripheral responses. Our results suggest that the α7nAchR might also regulate the response to local inflammation within the bone since it decreases IL1β-induced IL6 and MMP expressions *in vitro*. Interestingly, the decrease of IL1β-induced RANKL after nicotine treatment was not lost in α7nAChR KO OB, meaning that other nAchR might be involved in the subchondral remodeling process. In addition, we found that α7nAChR KO mice of 9 and 12 months had slightly more cartilage defect than WT but without any subchondral remodeling modification. This effect on cartilage might be due to an altered response of chondrocytes (16–18). However, our results can suggest that the loss of α7nAChR in OB also participates in cartilage defect in an inflammation context because of a lack of decrease in IL6 and MMP3. Articular cartilage is a unique tissue since it is neither innervated nor vascularized. Therefore, it communicates very closely with subchondral bone through microcracks and vascular channels that can facilitate the passage of MMP3 from subchondral bone to deep zone cartilage (26).

The absence of effect of α7nAChR on subchondral bone remodeling *in vivo* and its markers *in vitro* (i.e., OPG and RANKL) is concordant with previous findings. α7nAchR seems mostly involved in inflammation and catabolism rather than in bone remodeling. Previous evaluation of α7nAChR in bone involvement using Chrna7^-/-^ male mice showed no difference with WT in a microCT evaluation of all long bone volume (27). In contrast, female Chrna7^-/-^ mice had slight modification of their bone mass with increased stiffness and cortical thickness due to an increase in bone formation (28, 29). Here, *in vivo* analyses were performed in male mice because C57B6 male are usually considered as more sensitive to OA development than female (30). However, the cholinergic system seems to influence bone remodeling through other receptors like alpha 9 nicotinic subunit or muscarinic M3 receptors (27, 31).

Finally, α7nAChR activation has shown very encouraging *in vitro* and *in vivo* results towards inflammation and neurological processes in mice. In human, a specific duplicate might mitigate the effect of the agonist. Our results confirm that nAchR of human outgrowth OB did not elicit anti-inflammatory or anticatabolic responses, conversely to murine OB. This could be due to the CHRFAM7A duplicate expressed by human OB since the presence of CHRFAM7A decreases agonist binding in human cells (32). Almost all the population carries the CHRFAM7A duplicate but a 2-base-pair deletion in CHRFAM7A, highly present in the population, is considered as a null allele for CHRFAM7A. Indeed, approximatively 25% is a non-carrier of CHRFAM7A and might benefit the α7nAChR agonist (33). Another explanation is the possible presence of other nicotinic receptors in human OBs because they expressed higher levels of chrna5 and chrnb4 than murine OB. These subunits could assemble and modify agonist response.

This study has some limitations. First of all, it is not possible to directly extract mature OB from human subchondral bone. We therefore worked on human OBs derived from subchondral bone precursors, as differentiation may have altered the expression of some markers of the cholinergic system. However, this is a previously published and used cell culture protocol and we demonstrated that the differentiation was functionally effective since these cells calcified the extracellular matrix. Furthermore, we pooled both sclerotic and non-sclerotic areas of subchondral tibial plateaus for OB culture while the expression of cholinergic markers could be different between these 2 zones and should be compared since there is intrapatient differences in gene expression profiles and mineralization capacities between these 2 zones (34–36). Finally, the assessment of bone mass is more accurate *via* microCT than by measurement of bone areas.

In conclusion, this study has shown that human and murine OBs belong to the non-neuronal cholinergic system. α7nAChR activation decreased inflammatory and catabolic responses of IL1b-OB activated in mice and might be involved in cartilage homeostasis during aging, while it has no effect on bone remodeling. In human outgrowth OB, the role of the cholinergic system might be restrained depending on the CHRFAM7A genotype.

## Data Availability Statement

The raw data supporting the conclusions of this article will be made available by the authors, without undue reservation.

## Ethics Statement

The studies involving human participants were reviewed and approved by Comité de Protection des Personnes, Paris Ile de France 5 and Commission Nationale de l’Informatique et des Libertés. The patients/participants provided their written informed consent to participate in this study. The animal study was reviewed and approved by Comité National de Réflexion Ethique sur l’Experimentation Animale (Ile-de-France, Paris, No. 5).

## Author Contributions

AC, JP, AD, ML, IK, AP, PS, F-PE, FB, and JS have made substantial contributions to the conception and the design of the work, and the acquisition, analysis, and the interpretation of data. AC and JS have drafted the manuscript. All authors contributed to the article and approved the submitted version.

## Funding

AC and IK have been funded by Société Française de Rhumatologie; AC and JP have been funded by Assistance publique – Hôpitaux de Paris; PS has received an EMBO grant.

## Conflict of Interest

The authors declare that the research was conducted in the absence of any commercial or financial relationships that could be construed as a potential conflict of interest.

## Publisher’s Note

All claims expressed in this article are solely those of the authors and do not necessarily represent those of their affiliated organizations, or those of the publisher, the editors and the reviewers. Any product that may be evaluated in this article, or claim that may be made by its manufacturer, is not guaranteed or endorsed by the publisher.
